# Future expenditure risk of silent members: a statistical analysis

**DOI:** 10.1186/s12913-016-1552-x

**Published:** 2016-07-29

**Authors:** Trudy Millard Krause, Elifnur Yay Donderici, Cecilia Ganduglia Cazaban, Luisa Franzini

**Affiliations:** 1 Department of Management, Policy and Community Health, University of Texas School of Public Health, Co-Director, UTSPH – Center for Healthcare Data Research, University of Texas School of Public Health, University of Texas School of Public Health, 1200 Pressler Street RAS E905, Houston, TX 77030 USA; 2 Department of Management, Policy and Community Health, University of Texas School of Public Health, 1200 Pressler Street RAS E345, Houston, TX 77030 USA; 3 Department of Health Services Administration, School of Public Health, University of Maryland, 3310D SPH Building #255, College Park, MD 20742-2611 USA

**Keywords:** Care-avoidance, Silent-members, Risk, PMPY, Claims-data, Expenditures

## Abstract

**Background:**

Silent-members are members of a medical health plan who submit no claims for healthcare services in a benefit year despite 12 months of continuous-enrollment. This study was conducted to evaluate the future expenditure risk of commercial-insured members who avoid all medical care despite coverage. In order to determine if the silent-members were at greater risk, we compared them to members who received care in the anchor year (2009) but had low-expenditures. The low-expenditure members were assumed to represent persons without significant medical conditions and without care-avoidance behaviors. We examined the claims experience of a cohort of silent members in the 2 years after the silent year (2009) and compared it with the corresponding claims experience for a cohort of low-expenditure members from the same anchor year (2009).

**Methods:**

Members of commercial health plans (BCBS of Texas) were selected based on continuous-enrollment in 2009. Two sub-groups were identified based on annual claims expenditure: Care avoiders were members with 12 months continuous-enrollment and no medical claims, and are thus referred to as “silent members” in the insurance industry. Low-Expenditure members were those with 12 months continuous-enrollment and total PMPY (per member per year) annual medical claims expenditure in the lowest 10th percentile of members with claims experience. “Low-expenditure” members served as a comparison group to the “silent members”, under the assumption that such claimants were using benefits for minor healthcare issues as needed. Key variables were enrollment and expenditures. Enrollment data identified demographics and continuous-enrollment. Medical claims data were used to calculate utilization and expenditures. All claims data were de-identified and no consent was required, as approved by the Institutional Review Board. No research involved human subjects. Multivariate logistic regression models were applied.

**Results:**

Silent members who seek care in subsequent years have a greater probability of becoming high-expenditure claimants than those with low-expenditure experience.

**Conclusions:**

For silent members who subsequently seek treatment, the probability of becoming high-expenditure is significantly greater than low-expenditure members from the anchor year. The implications of future high costs for silent members who become claimants may support the need for additional research to address the risks of care avoidance behaviors.

## Background

Care avoiders are persons who do not seek medical care or treatment despite suspected symptoms or health complaints, often due to cost or beliefs. However, some care avoiders simply chose to avoid the medical system, despite available coverage, appropriateness for recommended prevention services, and access. These individuals are referred to as “silent-members” within the benefits industry. Silent-members are members of a medical health plan who submit no claims for healthcare services in a benefit year despite 12 months of continuous-enrollment. These members appear desirable to a commercial carrier for their apparent low risk due to their lack of expenditures. Yet, these members are avoiding routine care to detect asymptomatic conditions, medical management, or preventive care and recommended screenings. Lack of claims data on these silent-members prevents the health plan from assigning a risk value to them for purposes of plan risk adjustment and reporting purposes. Thus, their future risk remains unknown until the point of utilization.

Previous studies of care-avoiders have largely focused on the avoidance of care despite warning signs of disease, such as cancer and heart disease [1–3]. Most studies have found a variety of reasons for avoiding medical care, such as socio-demographic factors, personal belief factors, provider factors (distrust, access), or cost factors [4–9].

Silent-members of commercial health plans may be motivated by any of the offered reasons for care-avoidance. Cost related issues for care-avoidance are unlikely within the commercial population, which covers mostly employed persons and their dependents. However, employer-sponsored health plans often offer a benefit plan that shifts initial costs to the member. Several studies have assessed the impact of these high-deductible-health-plans or consumer-driven-health-plans (CDHP) on care-avoidance. The Actuaries Study of 2009 reviewed studies comparing CDHP plans to traditional plans and concluded that CDHP members had higher rates for preventive services, most likely due to the fact that much preventive care is provided at no cost to the member [10]. It also noted that 3 of the 4 studies found that CDHP members received recommended care for chronic conditions at the same or higher rate than the traditional plan members [11].

This study specifically targets silent-members to evaluate the risk of future high expenditures once they seek medical care. In order to determine if the silent-members were at greater risk, we compared them to members who received care in the anchor year (2009) but had low-expenditures. The low-expenditure members were assumed to represent persons without significant medical conditions and without care-avoidance behaviors. We examined the claims experience of a cohort of silent members in the two years after the silent year (2009) and compared it with the corresponding claims experience for a cohort of low-expenditure members from the same anchor year (2009). Silent-members were identified in the anchor year 2009 as members enrolled for 12 months who submitted no claims for medical benefits. The low-expenditure members were identified as members whose annual expenditure in 2009 was within the 10th percentile of all members. To determine the ranges of per member annual expenditures, the average annual per-member-per-year amount (PMPY) was computed. Due to data limitations that did not define plan design, we were unable to study the impact of a CDH benefit design on silent-members, but assumed that the impact would be the same on the low-expenditure members. Due to the studies mentioned previously, we concluded that plan design and cost issues were not drivers for care-avoidance in the commercial population. We evaluated the impact of multiple demographic and socioeconomic factors available in claims data, such as age, gender, and socioeconomic at the residential zip code level.

We compared the probability of silent-members becoming high expenditure in each of the following two years (2010, 2011) and the combined 2 year period with the corresponding probability in the low-expenditure member comparison group.

## Methods

Enrollment and medical claims data were obtained from Blue Cross Blue Shield of Texas (BCBSTX), which covers approximately a third of the commercial population in the state. All claims data were de-identified at the source, and no consent was required, as approved by the Institutional Review Board. No research involved human subjects. The BCBSTX members within the dataset are members of preferred provider organizations (PPO). Approximately 95 % of BCBSTX members are in PPO plans. Only members, age 18 to 63 (in 2009), with a zip code in Texas, and with twelve months continuous-enrollment in 2009 were included in the study. This provided a population of 1,319,240 adult members in 2009.

Using the medical claims file, we computed annual medical claims expense per member per year (PMPY), which consists of expenditures for inpatient hospital care, outpatient facility services, and professional services. Claims expense was defined by the total allowed amount which includes any deductible, co-pay, and coinsurance paid out-of-pocket by the patient, as well as payments made by BCBSTX to providers. PMPY expenses were estimated by dividing the total annual expenditures for medical claims by the total count of members with continuous enrollment. Thus, individual member PMPY amounts can be distributed in relation to the mean.

Silent-members were identified as those members (with 12 months continuous-enrollment in 2009 in the enrollment file) with no medical claims submitted in 2009. Claims with a 0 or negative payment amount were considered to be submitted claims for service, however this represented a minimal number of claims. The comparison group was identified as “low-expenditure” members, under the assumption that such claimants were using benefits for minor healthcare issues thus obviating several avoidance factors. To identify this cohort, we computed PMPY expenditures for the full population and identified percentiles of expenditure amounts. The “low-expenditure” members were identified as those members whose mean annual expenditure was within the 10th percentile. The most frequent reasons “low cost members” used healthcare services were routine gynecological examinations and routine general examinations as well as acute episodes, most commonly upper airway infections. In 2009 the mean PMPY for all members with 12 months continuous-enrollment was $3,645, and the 10th percentile for PMPY expense was $148.

In order to evaluate differences between silent-members and low-expenditure members, we assessed the impact of age, gender, and socioeconomic variables. Age, gender and zip code variables were included in the BCBSTX enrollment data. However, claims data and the related enrollment data did not include member socioeconomic variables. Therefore, socioeconomic status variables of race, education and income were generated using the information in the American Community Survey (ACS) data for year 2010 [11]. We used the 5-digit zip code registered for the member in the enrollment file to generate a crosswalk with the zip code tabulated area information recorded in the ACS. We then imputed the ACS statistics to the members in that zip code.

Silent-members and low-expenditure members from 2009 were tracked in 2010 and 2011 to identify future claims expenditures. We followed each member according to different continuous-enrollment requirements in 2010 and 2011: a minimum of 13 month continuous-enrollment (12 months in 2009 and 1 month in 2010); a minimum of 18 months continuous-enrollment (12 months in 2009 and 6 months in 2010), 24 months continuous-enrollment (12 months in 2009 and 12 months in 2010), and 36 months enrollment (12 months in each 2009, 2010 & 2011). The various enrollment spans were reviewed in order to capture individuals with high expenditures but who may not have survived or otherwise discontinued membership in the plan. High expenditure claimants had expenditures in 2010-2011 that fell into 1 of 3 levels. The first level consisted of members whose annual expenditures were within the top 5 % PMPY of all members ($15,193 in 2010, $15,780 in 2011, and $23,913 2010-2011). The second level threshold was set as within the top 1 % of PMPY ($54,542 in 2010, $56,496 in 2011, and $81,683 2010-2011) of all members, and the third level threshold was a PMPY greater than $100,000.

We conducted our analysis in 3 stages. First we measured and compared demographic characteristics between the two cohorts in 2009; silent-members and low-expenditure members. Continuous variables were compared using t-tests and binary variables using chi-square statistical tests. In addition to the elements in Table 1, we compared the distribution of silent-members to the low-expenditure members by zip code and found no significant difference.

**Table 1:** Baseline demographic and socio-economic characteristics

Demographic Characteristics	SILENT-MEMBERS 2009	LOW-EXPENDITURE MEMBERS 2009
Number of members	*238*,*180*	*121*,*700*
Male (%)^a^	158,589 (66.57 %)	73,815 (60.65 %)
Age group		
* Age 18*–*30*	70,281 (29.51 %)	35,748 (29.37 %)
* Age 30*–*40*	54,917 (23.06 %)	29,680 (24.39 %)
* Age 40*–*50*	59,998 (25.09 %)	28,478 (23.40 %)
* Age 50*–*63*	52,984 (22.25 %)	27,794 (22.84 %)
Race (% White)^a,b^	78.38	80.17
Education (% Less than high school degree)^a,b^	18.33	17.25
Income (% Below poverty line)^a,b^	16.30	15.45

^a^All comparisons were significant with *p*-values <0.0001

^b^Race, education and income were estimated using zip code level data

Secondly we estimated the odds of silent-members becoming high cost members using low-expenditure members as reference. Odds ratios were estimated using multivariate logistic regression models adjusting for gender, age and socio-demographic characteristics. The dependent variable was the likelihood of becoming a high expenditure claimant. To test the robustness of our results we ran different models accounting for different lengths of minimum enrollment as described above. We also evaluated different cutoff points for high-expenditure definition (top 1 percentile, top 5 percentile and over $100,000 in 24 months). Socio-demographic variables considered in the model included percent non-white, and percent less than high school education and median home value at the zip code level. These last 3 variables were highly correlated and therefore only the education level variable was included in the model. The same regression models were run stratifying by gender. Significant differences were determined when p values were less than 0.05.

We further evaluated the silent-members in subsequent years by those that (1) remained silent in 2010, 2011, and 2010-2011, compared to those that (2) broke silence and had a claim expense in 2010 or 2011. These 2 sub-groups of silent-members were then compared to the 2009 low-expenditure members who (1) became silent in 2010 or 2011 and (2) those that continued to have claims experience. Finally we estimated the mean difference in expenditures between groups using a log transformed linear regression model. A smearing factor was estimated to retransform the log scaled results into actual dollar amounts.

The group of silent-members and low-expenditure members who became excessively high-expenditure within two years, as defined by exceeding $100,000 in medical claims were studied in detail to determine the medical conditions responsible for the expense.

## Results

The population consisted of 1,319,240 members meeting the age and residency criteria who had 12 months continuous-enrollment in 2009. Silent-members numbered 238,180, which was 18.05 % of the members with continuous-enrollment in 2009. There were 121,700 low-expenditure members in the same year, which accounted for 11.3 % of continuously enrolled members. Comparison of the demographic and socio-economic status of both groups did not reveal considerable differences as shown in Table 1, though given the size of both cohorts statistical comparisons were significant at a 0.05 level. Younger men appeared to be slightly more likely to be silent members.

**Fig. 1:**
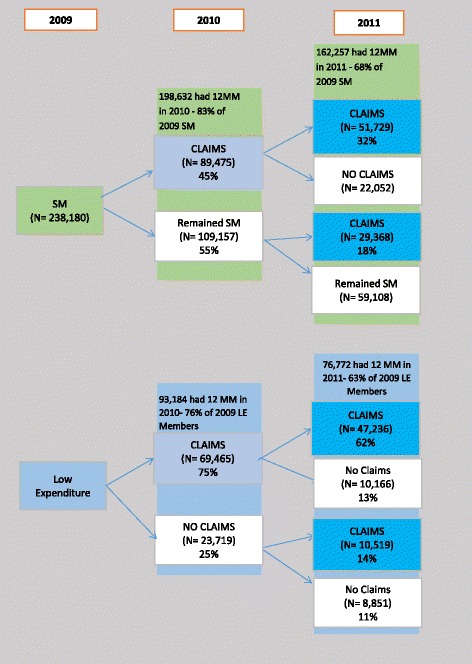
Experience of 2009 silent members and low-expenditure members in 2010 and 2011

Figure 1 illustrates the pattern of benefit use among the 2009 cohorts across the 2 subsequent years. Nearly 25 % of the 2009 silent-members remained silent across the subsequent two years (2010-2011), thus having a total of 36 months of no claims activity. Yet, only 7 % of the remaining 2009 low-expenditure members had no claims in 2010 and 2011. Of the 2009 silent-members who had claims in 2010, 29.5 % of those who remained in 2011 returned to silence in 2011.

The most conservative analysis compared silent-members of 2009 to low-expenditure members of 2009, requiring both groups to have 12 months continuous-enrollment in 2009, 2010 and 2011. In this analysis, each member had continuous 12-months enrollment within the plan for each of the three years, totaling 36 months enrollment. The results are shown in Table 2. It is interesting to note that the silent-members in 2009 had a higher rate of retention in the health plan in 2010 than that of low-expenditure members, yet the 2 year retention rate became somewhat closer between the two cohorts in the third year, 2011.

**Table 2:** Review requiring 12 member months continuous enrollment per year

	Count	Retention Rate	Count who had claims	Count who were Silent	PMPY Mean for cohort^a^ and for claimants only	PMPY Median for cohort^a^ and for claimants only	% > Top 5 % PMPY of All Members with 12 MM	% > Top 1 % PMPY of All Members with 12 MM	% > $100,000
Silent-members in 2009	238,180		0						
2010: 2009 Silent-members with 12 MM in 2010	198,632	83.40 %	89,475	109,157	Cohort^a^ $1,116	Cohort^a^ $0	1.22 %	0.26 %	0.10 %
					Claimants $2,477	Claimants $398	(*n* = 2432)	(*n* = 509)	(*n* = 205)
2011: 2009 Silent-members with 12 MM in 2010 and 2011	162,257	68.12 %	81,097	81,160	Cohort^a^ $1,481	Cohort^a^ $0	1.61 %	0.37 %	0.18 %
					Claimants $2,963	Claimants $439	(*n* = 2619)	(*n* = 607)	(*n* = 284)
	Count	Retention Rate	Count who had claims	Count who became silent	PMPY Mean	PMPY Median	% > Top 5 % PMPY of All Members with 12 MM	% > Top 1 % PMPY of All Members with 12 MM	% > $100,000
Low-Expenditure Members in 2009	121,700		121,700		$94.01	$95.27			
2009 Low-Expenditure Members with 12 MM in 2010	93,184	76.57 %	69,465	16,147	Cohort^a^ $1,462	Cohort^a^ $215	1.50 %	0.25 %	0.09 %
					Claimants $1,962	Claimants $383	(*n* = 1401)	(*n* = 231)	(*n* = 85)
2009 Low-Expenditure Members with 12 MM in 2011	76,772	63.08 %	57,755	12,298	Cohort^a^ $1,590	Cohort^a^ $150	1.92 %	0.35 %	0.17 %
					Claimants $2,466	Claimants $433	(*n* = 1472)	(*n* = 268)	(*n* = 129)

^a^Cohort refers to the group of members in the reported year who originated in 2009 and for whom PMPY means were estimated using all individuals originally assigned to the silent-member or low-expenditure member cohort in 2009, regardless of whether they had claims in that particular year. Persons for whom mean PMPY estimations were calculated using only those individuals from the cohort who had claims during the specific year are referred

We first compared the members that had at least one claim in 2010 or 2011. The logistic regression for 2009 silent-members who became claimants in 2010 revealed that silent-members with claims in 2010 were 1.66 times more likely to have 2010 annual expenditures in the top 1 percentile of all plan members than the 2009 low-expenditure members (Table 3). Additionally, they were 1.34 times more likely to have 2010 annual expenditures in the top 5th percentile than the 2009 low-expenditure members. The mean annual expenditure for 2009 silent-members who became claimants in 2010 was $2,477 compared to $1,962 for 2009 low-expenditure members (Table 2).

**Table 3:** Adjusted odds ratio for future high-expeniture and the incremental expenditure for 2009 silent members

	Cohort^a^			Claimants^b^		
	Odd Ratio^*^	95 % Confidence Interval		Odd Ratio^*^	95 % Confidence Interval	
Top cost percentile during 2010						
Top 1 Percentile	0.99	0.84	1.15	1.66^c^	1.42	1.94
Top 5 Percentile	0.8^c^	0.74	0.85	1.34^c^	1.25	1.43
Incremental Effect on PMPY	-$323.08	-$345.54	-$278.07	$494.72	$461.63	$526.01
Top cost percentile during 2011						
Top 1 Percentile	1.17^c^	1.01	1.37	1.80^c^	1.55	2.10
Top 5 Percentile	0.96	0.89	1.02	1.47^c^	1.37	1.57
Incremental Effect on PMPY	-$88.22	-$133.59	-$42.84	$831.80	$763.38	$879.21
Top cost percentile during 2010 and 2011 combined						
Top 1 Percentile	1.11	0.97	1.27	1.60^c^	1.40	1.84
Top 5 Percentile	0.89^c^	0.84	0.95	1.29^c^	1.25	1.41
Incremental Effect on PMPY	-$411.44	-$491.53	-$348.55	$647.10	$570.87	$703.56

^a^Cohort refers to the group of members in the reported year who originated in 2009 and for whom Odds Ratio were estimated using all individuals originally assigned to the silent-member or low-expenditure member cohort in 2009, regardless of whether they had claims in that particular year. Persons for whom Odds ratios were calculated using only those individuals from the cohort who had claims during the specific year are referred to as “Claimants”

^b^Odds ratios were adjusted by gender, age, percent non-white and percent with less than high school degree

^c^ *p*-value < 0.05

These cohort differences increased in the third year. In 2011, the 2009 silent-member claimants were 1.80 times more likely than the low-expenditure claimants to have annual expenditures in the top 1 percentile and 1.53 times more likely to be in the top 5 percentile (Table 3). The 2011 mean annual expenditure was $2,963 for 2009 silent-members and $2,113 for 2009 low-expenditure members (Table 2).

## Discussion

In reviewing expenditures spanning 2010 and 2011, it was noted that 40 % of 2009 silent-members who remained in the plan through 2011 also remained “silent”, and had no claims expense. Across the 24 months following the year of silence, 2009 silent-members who had claims in subsequent years were 1.60 times more likely to have total claims expenditure in the top 1 percentile and 1.13 times more likely to be in the top 5 percentile than 2009 low-expenditure members (Table 3).

Similar analyses were conducted with the lower continuous-enrollment requirements of 13 months, 18 months, and 24 months. These analyses were done to account for high expenditure claimants who may not have survived a serious illness and therefore had less than 12 continuous member months in each of the subsequent years. However, overall results were similar to the original findings that used 12 months continuous-enrollment in all three years (36 months).

All analyses were adjusted for age and gender. Consistently across the different comparisons, older patients were significantly more likely to become top 1 % and 5 % spenders. Gender was not a significant predictor of becoming high cost members. When the analysis was done stratifying by gender we found very similar effects, with slightly higher odds among males.

All analysis was adjusted for age and gender. Consistently across the different comparisons made, older patients were significantly more likely to become top 1 % and 5 % spenders. Gender was not a significant predictor of becoming high cost members. The percent of non-whites and proportion of individuals with less than a high school degree in the residence zip code were associated with slightly higher odds of becoming high cost members only in 2010 and 2011 combined.

Results from the log transformed linear regression model (Table 3) showed that silent members who became claimants in subsequent years had higher expenditures than low-expenditures members (incremental effect on yearly expenditure for 2010: $494; 2011: $831; combined 2010 and 2011: $647). Table 3 shows as well that silent-members overall (whether they had claims or not in 2010 or 2011) were less expensive in subsequent years than low-expenditures members (negative incremental effect on yearly expenditures for 2010: -$323; 2011: $-57 and combined 2010 and 2011: $-411).

The groups of silent-members and low-expenditure members who become excessively high-expenditure within two years, as defined by exceeding $100,000 in medical claims were studied in detail to determine the medical conditions responsible for the expense. Both groups had a high proportion of cases over $100,000 in two years that were cancer-related expenditures (42 % of the silent-members and 44 % of the low-expenditure members). The two groups also had similar proportions attributed to injury (11 % of silent-members and 9 % of low-expenditure members), cardiovascular (15 % and 11 %), and musculoskeletal (6 % and 9 %).

When reviewed as a cohort of silent members regardless of future claims experience, (as reported in Table 2), fewer 2009 silent-members fell into the 2010 top 1 percentile (0.26 %) and the 2010 top 5 percentile (1.22 %) than the 2009 low-expenditure members (0.37 % and 1.61 % respectively). As a cohort, the 2010 mean annual expenditure for 2009 silent-members was $1,116 compared to $1,462 for 2009 low-expenditure members. In 2011, the 2009 silent-member cohort had a mean annual expenditure of $1,481 compared to $1,590 for 2009 low-expenditure members. And, in 2011 0.37 % of 2009 silent-members fell in the top 1 percentile compared to 0.35 % of the 2009 low-expenditure members, and 1.61 % compared to 1.92 % for the top 5 percentile. Therefore, as a complete cohort, the silent-members generally spent less per subsequent year and were generally less likely to become high-expenditure in subsequent years.

In summary, the silent-members that utilize healthcare resources in subsequent years have a significantly higher average annual spend than the low-expenditure counterparts, as well as a greater likelihood of becoming high expenditure claimants. There are no demographic differences between the 2 groups that might explain the observations. Conditions that lead to high expenditures remain similar between the 2 groups. In contrast though, the full silent-member cohort (inclusive of those that remain silent and those that become claimants), has a lower PMPY expenditure in subsequent years than the cohort of low-expenditure members. And, the silent-member cohort has a lower probability of becoming high cost members in subsequent years than a cohort of low-expenditure members.

### Study limitations

This study may be limited by the lack of various data variables within the claims data. For example, the data did not include pharmacy claims and the associated costs. This limitation may have prevented some members from reaching the defined limits of high expenditure claimants such as those with medical conditions that rely on costly specialty drugs. Furthermore, the claims within the dataset were provided by a single commercial carrier and may not be reflective of all commercial carriers.

Additionally, the claims were for residents of the State of Texas only and may not be reflective of residents of other states. The data did not provide a variable to identify high deductible plans, which might have been an influential factor for care-avoidance.

Finally, some silent-members, as well as low-expenditure members may have developed a potentially costly condition in the subsequent year (s) but may have either died or left the plan as a result of the condition before claims costs increased to the high-cost levels.

## Conclusions

This study serves as the initial step in observing the cost impact of silent-members in commercial health plans. The findings of this study may support the assumption that as a whole, silent-members are generally healthier and may possibly avoid care due to lack of need. However, once a previously silent-member seeks care, he/she is more likely to become high cost than members that have routinely received medical attention. Perhaps this indicates that medical care is sought by previously silent-members once symptoms become obtrusive, thus implying that earlier opportunities for identification and intervention were missed. The implications of future high costs for silent-members who become claimants may reinforce concern over the risks of care avoidance behaviors and the need for routine medical management. Long term studies of such members would be interesting to assess if cost risks change over years.

As health plans struggle to control costs, many efforts are directed toward medical management and wellness initiatives. Many of these initiatives target silent-members in the belief that care-avoidance may result in lost intervention opportunities and ultimately greater expense. This research is the first attempt to define and describe the implications of silent-members in commercial health plans.

Medical management strategies often include the assignment of a risk value to each plan member. Risk values are derived by data within the claims files. If members are silent and submit no claims for services, then a risk value cannot be assigned to that member, other than one derived from demographics. The Affordable Care Act has established a process for commercial plans operating small group plans and exchange plans to submit data in order to derive risk assessment scores for the population served. From this score, CMS (Center for Medicare and Medicaid Services) has created a policy of redistributing funds within a risk adjustment pool as an attempt to stabilize premiums and to mitigate the impact of potential adverse selection. Plans will recognize the need to identify clinical risk values of the 18 % of members who tend to be silent, and may soon take action to encourage silent members to break their silence and generate claims each benefit year that record diagnoses.

Policy implications for healthcare managers and health plan member relations include the need to question the concern about silent-members as a desirable phenomenon. Wellness initiatives and early intervention strategies for silent-members, although well intended may be justified for overall plan risk assessment purposes. General education, benefit awareness, and encouragement for ongoing, routine medical management may discourage care avoidance for acute symptoms, thus targeting silent-members who delay seeking care.

## Abbreviations

ACS, American Community Survey; BCBSTX, Blue Cross Blue Shield of Texas; CDHP, Consumer Driven Health Plan; CMS, Center for Medicare and Medicaid Services; PMPY, Per Member Per Year; PPO, Preferred Provider Organization
